# Identification of QTLs and candidate genes associated with seed germination capacity under heat stress in watermelon

**DOI:** 10.3389/fpls.2026.1932864

**Published:** 2026-10-30

**Authors:** Jianfei Song, Yiying Zhang, Nannan Li, Jianli Shang, Dan Zhou, Shuangwu Ma, Na Li

**Affiliations:** 1 Zhengzhou Fruit Research Institute, Chinese Academy of Agricultural Sciences, Zhengzhou, Henan, China; 2 National Nanfan Research Institute (Sanya), Chinese Academy of Agricultural Sciences, Sanya, Hainan, China

**Keywords:** heat stress, QTL, seed germination capacity, SNP, watermelon

## Abstract

**Background:**

Heat stress (HS) severely inhibits seed germination of direct-seeded crops such as watermelon. However, the genetic basis governing germination capacity under HS remains largely unclear.

**Methods:**

An F2 population consisting of 132 individuals was developed from a cross between the heat-tolerant inbred line 8R098 (high germination under HS) and the heat-sensitive line 8R122 (low germination under HS). SNP-trait association analysis and linkage mapping were employed to dissect the genetic basis of seed germination capacity under HS. Germination-stage RNA-seq of high- and low-germination accessions under HS was performed to screen the DEGs.

**Results:**

Heat tolerance for seed germination segregated at a high-to-low ratio of 1: 3, indicating a nearly recessive genetic nature. A major QTL for HS germination tolerance was fine-mapped to the interval of 29.05~33.41 Mb on chromosome 10, harboring 472 annotated genes. RNA-seq identified 1,519 HS-responsive DEGs enriched in plant hormone signal transduction and metabolic pathways. A total of 101 putative functional non-synonymous SNPs (nsSNPs) were found in 76 protein-coding genes within this QTL region. Integrative analysis of DEGs and nsSNP-containing genes, followed by filtering genes with inconsistent expression trends, retained four candidate genes with allelic variations, namely *Cla97C10G205480, Cla97C10G205850, Cla97C10G204760*, and *Cla97C10G203920*.

**Conclusion:**

Our findings reveal candidate genes regulating thermotolerance during watermelon seed germination and provide useful genetic resources for breeding watermelon varieties with improved heat tolerance at the germination stage.

## 1 Introduction

With rising global temperatures, heat stress (HS) has become a major environmental constraint on crop production (Zheng et al., 2025). HS not only diminishes photosynthetic efficiency and perturbs metabolic homeostasis in plants, but also induces leaf wilting and necrosis (Mathew et al., 2025; Zheng et al., 2025), ultimately reducing yield and quality (Lesk et al., 2022). Seed germination is the key initiation stage of the plant life cycle and is particularly susceptible to temperature. Under HS, crops frequently undergo thermal dormancy or growth arrest, leading to diminished germination vigor and failure to germinate normally (Reed et al., 2022). In addition, HS during germination exerts adverse effects on seed quality, dormancy, germination, emergence, and seedling establishment (Finkelstein et al., 2008; Brunel-Muguet et al., 2015), thus impairing seed viability and germinative capacity (Fahad et al., 2017). Consequently, breeding heat-tolerant crop varieties is imperative to mitigate the impacts of the global climate crisis.

Seed germination capacity is an important agronomic trait governed by both maternal tissues (seed coat and endosperm) and the embryo genotype. The maternal tissues determine initial vigor and physical barriers, whereas the embryo’s stress-response pathways are critical for successful germination under HS (Oh et al., 2025; Gu et al., 2014). Several quantitative trait loci (QTLs) and genes regulating temperature-mediated germination have been identified in other crops. In rice, the OSK3-OsbZIP58 and the OsFTIP9-OSH1 modules modulate germination under temperature stress (Xu et al., 2026; Zhang et al., 2025). In lettuce, *Htg9.1* and *NCED4*-mediated abscisic acid (ABA) biosynthesis are key to high-temperature inhibition (Huo et al., 2013; Yoong et al., 2016). By contrast, most existing studies have focused on physiological characterization of germination thermotolerance (Zhou et al., 2018), whereas systematic QTL mapping and candidate gene mining based on natural variation are rarely reported.

Watermelon (*Citrullus lanatus*), a widely cultivated fruit crop worldwide, flourishes at temperatures ranging from 20 to 32°C (Fierascu et al., 2020; Guo et al., 2013; Tian et al., 2018). Excessively high temperatures markedly reduce its seed germination rate (Guan et al., 2020). However, the genetic loci regulating watermelon germination capacity under HS have yet to be identified. In this study, we integrated single-nucleotide polymorphism (SNP)-trait association analysis, quantitative trait locus (QTL) mapping, and transcriptome sequencing (RNA-seq) to identify a key genetic locus associated with seed germination ability under HS, providing gene resources for improving heat-tolerant germination capacity in watermelon.

## 2 Materials and methods

### 2.1 Plant materials

The heat-tolerant line 8R098 and heat-sensitive 8R122 were obtained from the National Mid-term Genebank for Watermelon and Melon (Zhengzhou, China). An F_2_ population of 132 individuals was developed from the cross between 8R098 and 8R122. Parental, F_1_, and F_2_ plants were grown in a greenhouse under standard agronomic practices. Fruits were harvested 45~50 days after manual pollination.

### 2.2 Stress treatment

Germination assays included control and HS treatments. Based on a preliminary experiment and our previous study (Zhou et al., 2018), 33°C and 42°C were chosen as control and stress temperatures, respectively. Given that germination performance under control conditions has been preliminarily validated with an inter-replicate coefficient of variation <5%, and that HS is the main source of effect in this study, three replicates for parental lines and one replicate for individual F_2_ plants (5 or 10 seeds per replicate) were used under control conditions; nine replicates for parents and three replicates for F_2_ individuals were employed under heat stress (HS) (**Supplementary Table S1**). Germination was defined as radicle protrusion ≥1 mm and recorded every 24 h for 6 days. Germinated seeds were removed, and water was added as needed. Final germination percentage (GP), germination energy (GE), germination index (GI), relative germination percentage (RGP), and relative germination energy (RGE) were calculated as described in **Table 1**.

**Table 1:** Formulas for the calculation of germination indices.

Parameter	Formula for calculation	Description
GP	(N_p_/N_t_) × 100%	N_p_: number of germinated seeds; N_t_: total number of seeds
GE	(n_3_/Nt) × 100%	n_3_: number of seeds germinated on day 3
GI	∑G_t_/t	The number of newly germinated seeds on day t, where t represents the incubation day.
RGP	S_i_/K_i_	S_i_: germination percentage under stress; K_i_: germination percentage under control
RGE	S_3_/K_3_	S_3_: germination percentage under stress on day 3; K_3_: germination percentage under control on day 3

GP, germination percentage; GE, germination energy; GI, germination index; RGP, relative germination percentage; RGE, relative germination energy.

### 2.3 SNP-trait association analysis and QTL mapping

Genomic DNA was extracted from leaves of the two parents and 132 F_2_ individuals using the cetyltrimethylammonium bromide (CTAB) method. After quality assessment, 0.5 μg of DNA per sample was used to construct libraries with the TruSeq Nano DNA HT Sample Prep Kit (Illumina). Libraries were sequenced on an Illumina NovaSeq X Plus platform (paired-end, 150 bp) at Shanghai Majorbio Bio-pharm Technology Co., Ltd.

Raw reads were trimmed with fastp and aligned to the watermelon reference genome (97103v2) using BWA-MEM. Alignments were sorted, duplicates marked, and base quality recalibrated following GATK Best Practices. SNPs and insertions and deletions (InDels) were jointly called with Sentieon, filtered, and annotated with SnpEff. After linkage disequilibrium (LD) pruning with PLINK, principal component analysis (PCA) was performed, and a kinship matrix was computed. SNP-trait association analysis was performed using the general linear model (GLM) in ‘rMVP’ (Yin et al., 2021), with the Q matrix and the first three principal components (PCs) included as covariates to control for population structure. The genome-wide significance threshold was set at *P* = 0.05/(total SNPs). A genetic linkage map was constructed using JoinMap 4.0 (logarithm of odds [LOD] ≥ 2.0, recombination frequency < 0.4). Genetic distances were calculated with the Kosambi function. QTL analysis was conducted using composite interval mapping in WinQTL Cartographer, with significance thresholds determined by 1,000 permutation tests (*P* = 0.05).

### 2.4 Gene expression analysis

RNA-seq was conducted as previously described (Song et al., 2026). Briefly, germinated seeds at 48 h post-imbibition (radicle protrusion stage) were dissected to remove the seed coat, and the embryos were collected as material for transcriptome sequencing. A total of eight sample groups (parental lines and F_2_ extreme bulks, germinated at 33°C or 42°C for 48 h; **Table 2**) were analyzed, and each sample was set up with three biological replicates. Total RNA was extracted from de-hulled sprouting embryos. Libraries were constructed and sequenced by Beijing Biomarker Technologies Corporation. Clean reads were aligned to the watermelon reference genome (97103v2) using the BMKCloud platform. Transcript abundance was quantified as fragments per kilobase of transcript per million mapped reads (FPKM) using StringTie. Differential expression analysis was performed independently using both DESeq2 and edgeR; differentially expressed genes (DEGs) were defined as |log_2_(Fold change)| ≥ 1 and *adjusted P-value* < 0.05. Gene Ontology (GO) and Kyoto Encyclopedia of Genes and Genomes (KEGG) enrichment analyses were conducted with clusterProfiler using the hypergeometric test.

**Table 2:** Grouping of samples for RNA sequencing (RNA-seq).

Population	Phenotype	Temperature	Sample ID
Parental (N)	High-germination (H)	33 °C	NH33
		42 °C	NH42
	Low-germination (L)	33 °C	NL33
		42 °C	NL42
F_2_ Bulk (P)	High-germination (H)	33 °C	PH33
		42 °C	PH42
	Low-germination (L)	33 °C	PL33
		42 °C	PL42

For reverse transcription quantitative polymerase chain reaction (RT-qPCR), the total RNA was reverse-transcribed into first-strand complementary DNA (cDNA) using a reverse transcription kit (Takara, Japan). Subsequently, the RT-qPCR was carried out as previously described (Song et al., 2025). Each sample contained three biological replicates and three technical replicates. The expression level was calculated using the 2^− ΔΔCT^ method. The watermelon actin gene *Cla97C02G026960* was used as the internal control. All the primers were listed in **Supplementary Table S2**.

### 2.5 Statistical analysis

Data Processing System (DPS) was utilized to perform statistical analysis. Origin 2025 (OriginLab Corporation, Northampton, MA, USA) and Excel 2019 were used to draw figures. Pairwise comparisons were conducted with a two-tailed Student’s t-test (*P < 0.05, **P < 0.01) and Duncan’s multiple range test following one-way ANOVA (*P* < 0.05).

## 3 Results

### 3.1 The heat-tolerant germination capacity is controlled by recessive inheritance

To investigate the heat-tolerant germination capacity of two watermelon varieties, namely 8R098 and 8R122, the five seed germination-related indices (GP, GE, GI, RGP, and RGE) were used to evaluate the germination capacity of parental lines and their F_1_ population (8R098 × 8R122 and its reciprocal, 8R122 × 8R098) under HS. Among them, 8R098 exhibited the highest germination capacity with 0.64 ± 0.11 GP, 0.43 ± 0.07 GE, 10.1 ± 1.34 GI, 0.64 ± 0.11 RGP, and 0.43 ± 0.07 RGE under HS. However, the seeds of 8R122 and the above F_1_ population lost germination capacity under HS (**Table 3**).

**Table 3:** Germination phenotypes of the parental lines and F_1_ progeny.

Samples	GP	GE	GI	RGP	RGE
8R098	0.64 ± 0.11a	0.43 ± 0.07a	10.1 ± 1.34a	0.64 ± 0.11a	0.43 ± 0.07a
8R098♀ × 8R122♂	0.00 ± 0.00b	0.00 ± 0.00b	0.00 ± 0.00b	0.00 ± 0.00b	0.00 ± 0.00b
8R122	0.00 ± 0.00b	0.00 ± 0.00b	0.00 ± 0.00b	0.00 ± 0.00b	0.00 ± 0.00b
8R122♀ × 8R098♂	0.00 ± 0.00b	0.00 ± 0.00b	0.00 ± 0.00b	0.00 ± 0.00b	0.00 ± 0.00b

Statistically significant differences (*P* < 0.05) are indicated by different letters in each column (one-way ANOVA). 8R098♀ × 8R122♂: 8R098 was used as the female parent, and 8R122 as the male parent. GP, germination percentage; GE, germination energy; GI, germination index; RGP, relative germination percentage; RGE, relative germination energy. 8R122♀ × 8R098♂: 8R122 was used as the female parent, and 8R098 as the male parent.

Furthermore, the germination indices of seeds harvested from each single watermelon fruit in the F_2_ population showed a bimodal distribution, with 54.2% of individuals having a 6-day germination percentage below 0.1 and 22.14% having a rate of 1.0 (**Figure 1**). Furthermore, we defined individuals with RGP ≥ 0.6 under HS as the high-germination phenotype and those with RGP < 0.6 as the low-germination phenotype. The ratio of high- to low-germination individuals approximately fit the theoretical 1:3 ratio (χ^2^ = 0.646, P = 0.421), implying that the seed germination capacity under HS is a near-quantitative trait controlled by recessive inheritance. Given the continuous phenotypic distribution of the F_2_ population, we inferred that the trait is controlled by a major recessive gene with minor-gene effects, consistent with quantitative inheritance.

**Figure 1:**
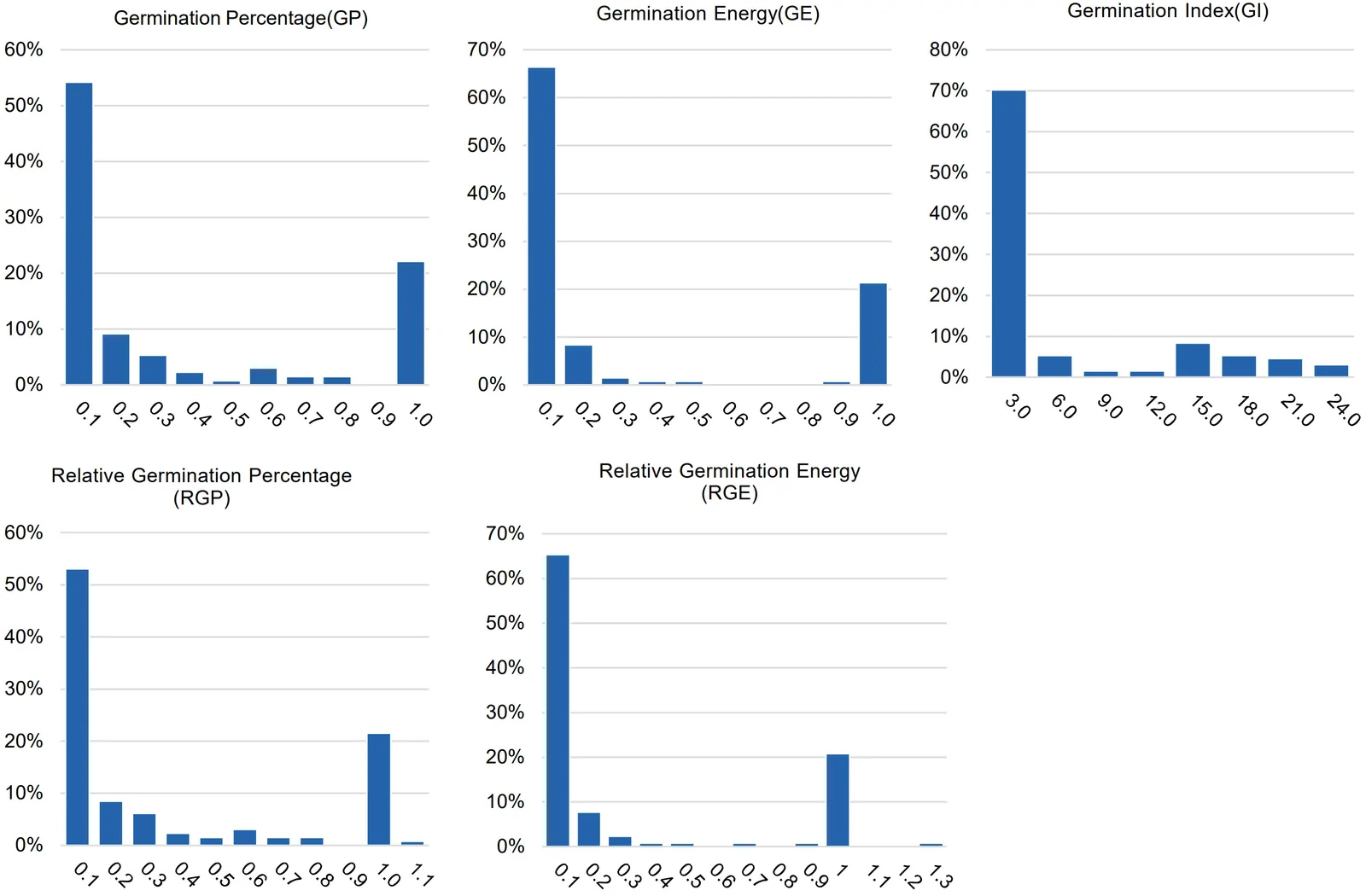
Frequency distribution of germination indices in the F_2_ population. All the indices showed a bimodal distribution.

### 3.2 Identification of major QTLs associated with heat-tolerant seed germination capacity

To identify genetic loci governing heat-tolerant germination capacity of seeds, an integrated analytical strategy was employed: genome-wide SNP-trait association analysis was first used for a whole-genome scan to preliminarily delimit candidate genomic intervals, followed by construction of a genetic linkage map for independent cross-validation of the locus mapping results. Through whole-genome resequencing of the two parental lines and their 132 F_2_ progeny individuals, a total of 617.26 Gb of high-quality clean data was generated. The average sequencing depth reached 30× for the parents and 13× for the F_2_ population, with a mean genome coverage of 95.62% and a guanine–cytosine (GC) content of 35.34% across samples (**Table S3**). Before downstream analyses, stringent quality filtering was applied to the SNP dataset to eliminate false positives based on four criteria: (i) removal of multi-allelic SNP loci; (ii) exclusion of loci with an average sequencing depth < 4 across all samples; (iii) removal of loci with a minor allele frequency (MAF) < 0.05; (iv) retention of only loci successfully genotyped in no less than 70% of individuals. Ultimately, a total of 350,803 SNPs and 61,561 InDels were retained for subsequent analyses.

Using GLM for association analysis, we identified a locus on chromosome 10 (28.05~34.80 Mb) as the most significant genomic region. Notably, this significant association signal was consistently detected across all five seed germination-related traits (**Figure 2**).

**Figure 2:**
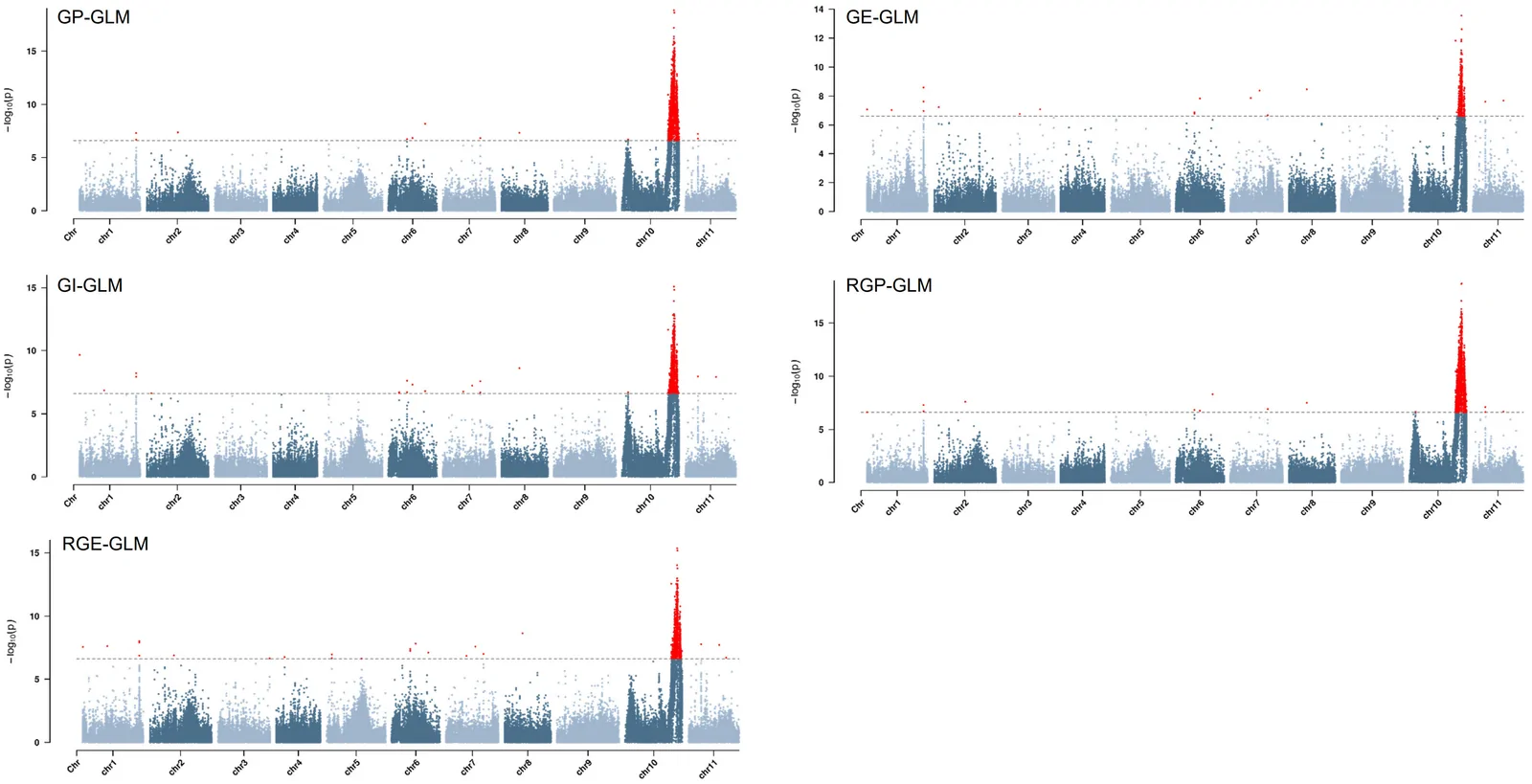
The genome-wide single-nucleotide polymorphism (SNP)-trait association analysis study of the germination capacity trait under heat stress. GP, germination percentage; GE, germination energy; GI, germination index; RGP, relative germination percentage; RGE, relative germination energy.

We also constructed a chromosome-specific genetic linkage map using 246 uniformly distributed markers on chromosome 10 to confirm the result of SNP-trait association analysis, which were curated from a total of 11,595 available SNPs, comprising both significantly associated variants and high-impact mutations (e.g., stop-gained, frameshift, and missense variants). The final linkage map spanned 321.40 cM (39.27 Mb), with an average inter-marker interval of 1.3 cM (0.16 Mb).

A QTL was mapped to the 29.05~33.41 Mb physical interval on chromosome 10 (**Figure 3**). This locus co-localized precisely with the SNP-trait association analysis signal, providing independent corroboration that this genomic locus modulates watermelon seed germination capacity under HS.

**Figure 3:**
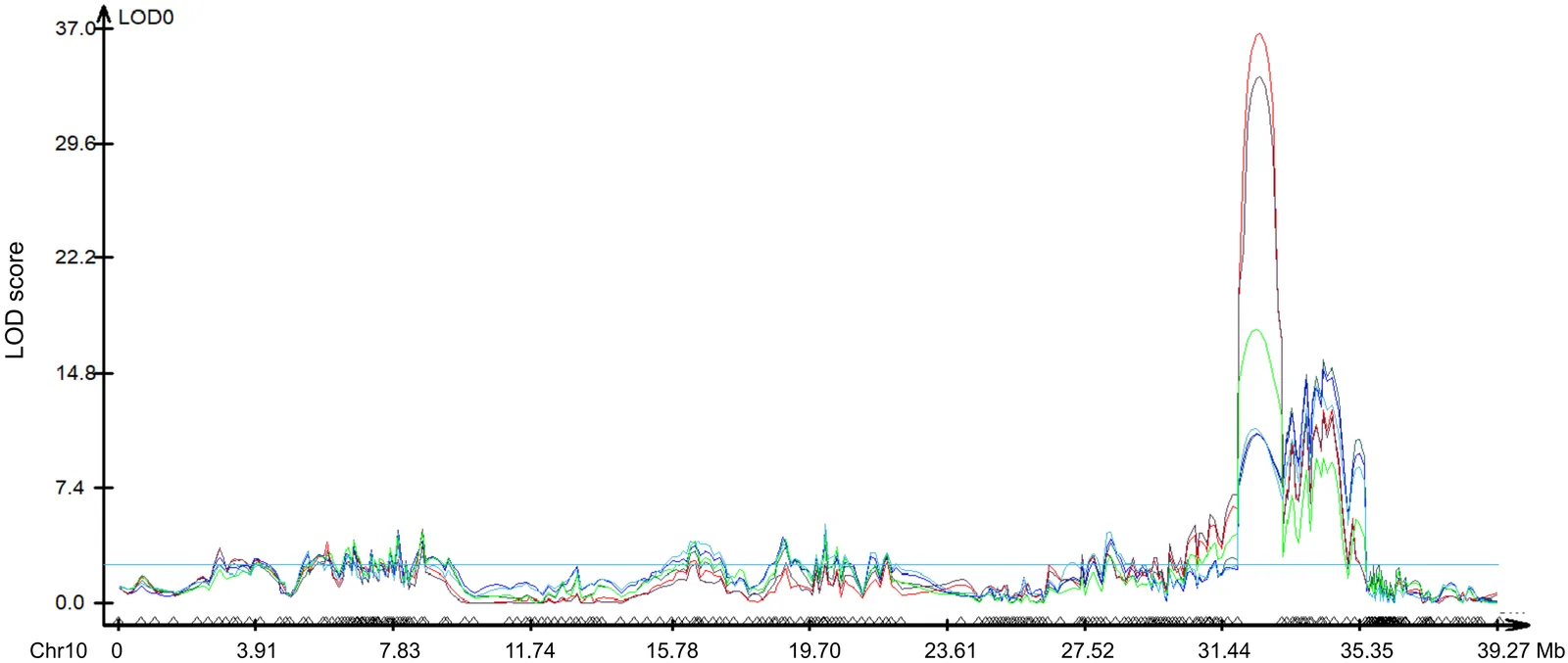
The quantitative trait locus (QTL) mapping of the germination capacity trait under heat stress on chromosome 10.

### 3.3 Identification of candidate genes within the major QTL via transcriptome analysis

To further screen potential genes regulating seed germination capacity, RNA-seq was conducted on eight sample groups, generating 164.28 Gb of clean data, with at least 5.92 Gb of clean data per sample and a minimum of 93.29% of bases with Phred quality scores ≥30 (Q30) (**Supplementary Table S4**). Because the candidate genes should respond to HS and exhibit differential expression between the high-germination and low-germination lines under HS, we conducted a Venn analysis of NL33 vs NL42, NH33 vs NH42, PL33 vs PL42, PH33 vs PH42, NL42 vs NH42, and PL42 vs PH42. A total of 1,519 common DEGs were identified (**Figure 4A**).

**Figure 4:**
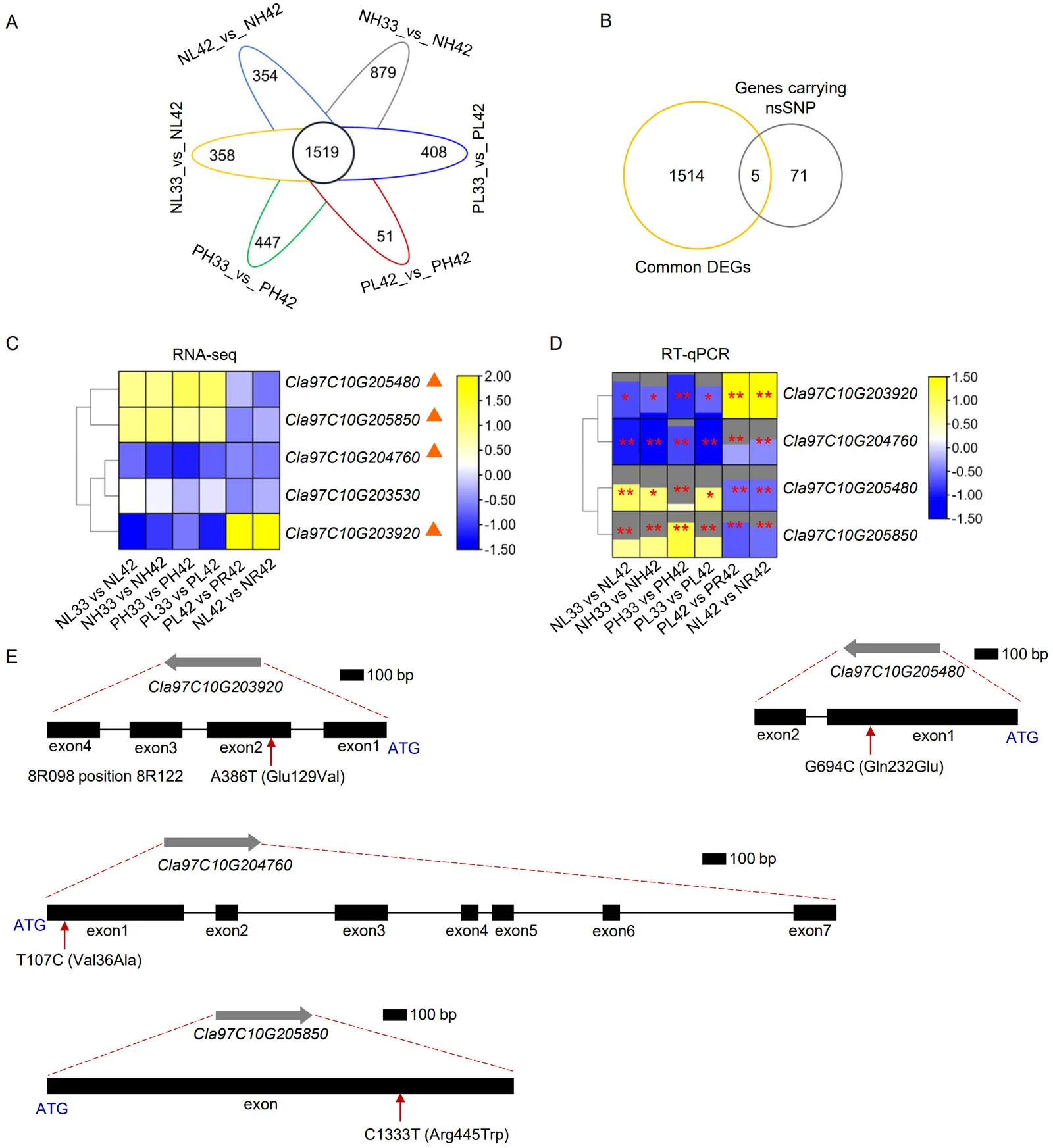
The identification of candidate genes associated with germination capacity under heat stress. **(A)** Petal Venn diagram showing the distribution of differentially expressed genes (DEGs) across six comparison groups, with 1,519 genes commonly differentially expressed in all groups. **(B)** Venn diagram illustrating the overlap between common DEGs and genes carrying non-synonymous single-nucleotide polymorphisms (nsSNPs), from which 5 candidate functional genes were identified. **(C)** Heatmap showing expression patterns of the 5 candidate genes in all comparison sets. The values were normalized by log_2_, and orange triangles label screened genes. **(D)** The RT-qPCR results of the identified four candidate genes. The values used for heatmap are ratios of relative expression between two samples, followed by row normalization. For NL33 vs NL42, the ratio is calculated as the relative expression level of a given gene in NL42 divided by its relative expression level in NL33. Yellow indicates up-regulation, and blue indicates down-regulation. Statistical significance was assessed using two-tailed student's t-test (*P < 0.05, **P < 0.01). **(E)** Gene structure and positions of non-synonymous SNPs (nsSNPs) in candidate genes. Black boxes indicate exons. ATG denotes the translation start site. Red arrows mark nsSNP positions, showing base substitutions and associated amino acid changes; 8R098 is on the left and 8R122 on the right. The black scale bar represents 100 bp.

Non-synonymous SNPs (nsSNPs) alter the structure of the encoded proteins and thereby modulate their biological functions (Yates and Sternberg, 2013). Here, from the 3,634 SNPs in the 29.05~33.41 Mb region of chromosome 10, a total of 101 putative functional nsSNPs were identified, which were mapped to 76 genes. Considering that candidate genes were required to (i) respond to HS (i.e., be differentially expressed between HS and control), (ii) show genotype-dependent expression differences between heat-tolerant and heat-sensitive parents and between the corresponding F_2_ extreme pools, and (iii) exhibit a directionally consistent expression pattern across these genotypes under HS, we combined the 76 genes and the 1,519 common DEGs and finally identified 5 genes, namely *Cla97C10G205480*, *Cla97C10G205850*, *Cla97C10G204760*, *Cla97C10G203920*, *Cla97C10G203530* (**Figure 4B**). However, *Cla97C10G203530* was excluded because it exhibited a different expression pattern in the PH33 vs PH42 comparison than in the NL33 vs NL42, NH33 vs NH42, and PL33 vs PL42 comparisons (**Figure 4C**). Furthermore, the RT-qPCR analysis confirmed the reliability of the transcriptome data (**Figure 4D**). The gene structures of candidate genes were also analyzed, and the positions of non-synonymous SNPs (nsSNPs) were mapped to exon regions (**Figure 4E**). Compared to the gene sequence of 8R098, the A386T variant of *Cla97C10G203920* in 8R122 caused a Glu129Val substitution. The G694C variant in *Cla97C10G205480* resulted in Gln232Glu. A T107C variant in *Cla97C10G204760* caused Val36Ala, while the C1333T variant in *Cla97C10G205850* produced Arg445Trp. All these nsSNPs reside within coding exons and introduce amino acid substitutions.

### 3.4 The identified candidate genes participate in heat stress responses via multiple distinct pathways

GO enrichment of the 1,519 DEGs assigned them to three categories, namely biological process (BP), cellular component (CC), and molecular function (MF). The top enriched terms were cellular and metabolic processes in BP, cellular anatomical entities and intracellular components in CC, and binding, catalytic activities, transport, and transcriptional regulation in MF (**Figure 5A**). KEGG enrichment grouped them into five main categories. Among these, plant hormone signal transduction was the single most enriched pathway, while metabolic pathways, including starch and sucrose metabolism, carbon metabolism, and amino acid biosynthesis, were the most abundant (**Figure 5B**).

**Figure 5:**
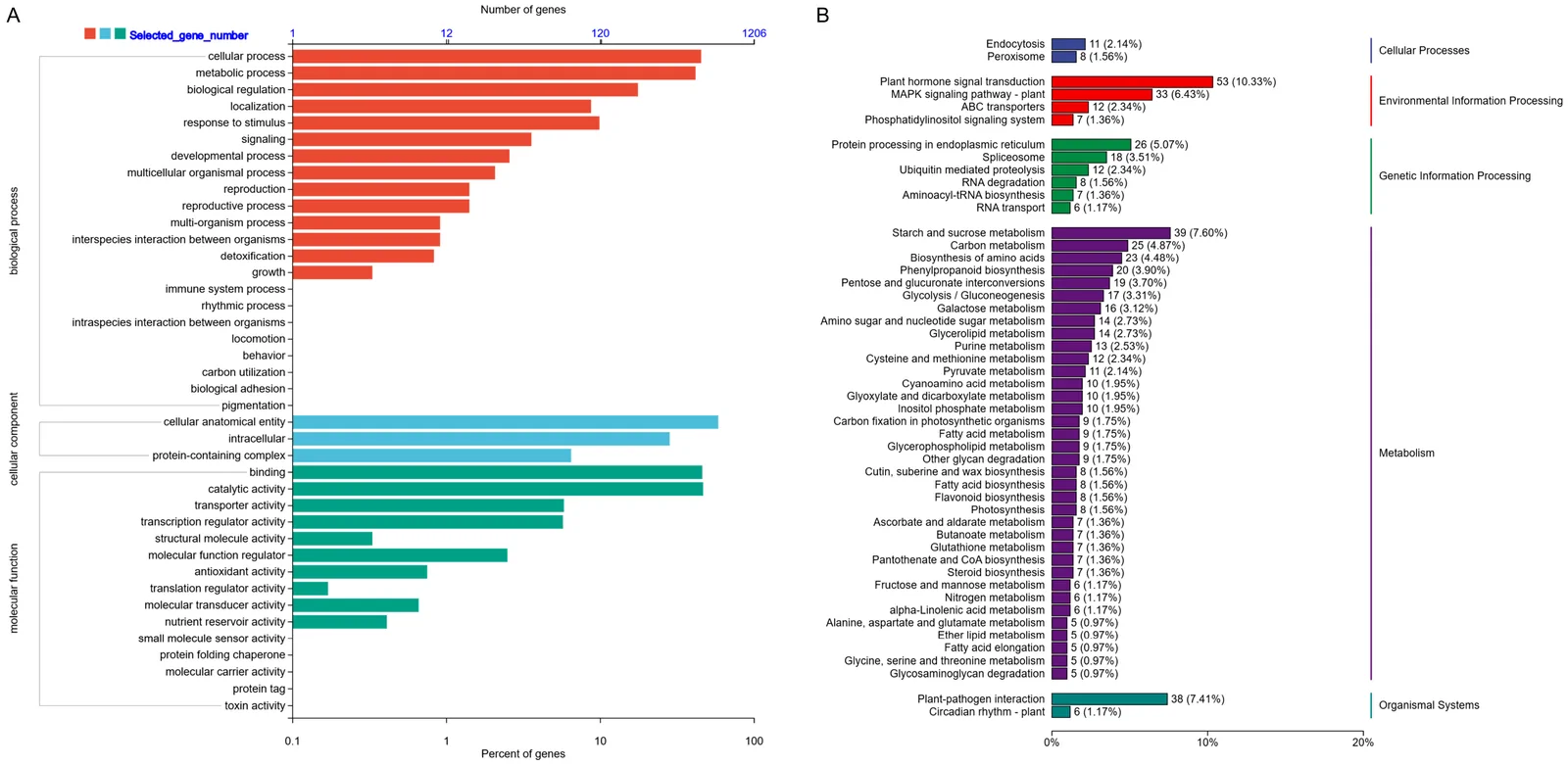
Gene ontology (GO) and Kyoto encyclopedia of genes and genomes (KEGG) functional enrichment of 1,519 common differentially expressed genes (DEGs). **(A)** Bar chart of GO classification, showing the proportion of enriched terms in biological process, cellular component, and molecular function. **(B)** Bar chart of enriched KEGG pathways, with primary pathway categories labeled on the right, gene counts and proportions indicated in parentheses.

Among the identified candidate genes, *Cla97C10G205480* is annotated as a VARLMGL domain-containing protein with limited functional information and carries a C-to-G mutation at nucleotide 694, causing a Glu-to-Gln substitution at residue 232. *Cla97C10G205850* encodes a putative pentatricopeptide repeat-containing protein and harbors a T-to-C mutation at nucleotide 1,333, resulting in a Trp-to-Arg change at residue 445. *Cla97C10G204760*, annotated as a chloroplastic-like RETICULATA protein that localizes to mitochondria and chloroplast membranes and may be involved in plant organ development, contains a C-to-T mutation at base 107, leading to an Ala-to-Val substitution at position 129. *Cla97C10G203920*, annotated as a putative protein phosphatase 2C with serine/threonine phosphatase activity and metal-ion-binding capability, potentially participating in signal transduction, carries a T-to-A mutation at base 386, resulting in a Glu-to-Val substitution at position 129. These results implied that the four candidate genes participate in HS responses via multiple distinct pathways (**Supplementary Table S5**).

## 4 Discussion

Seed germination is the initial stage of the plant life cycle and is affected by multiple factors, particularly temperature (Reed et al., 2022). HS severely impairs seed germination and constrains crop production, including watermelon (Karkute et al., 2021; Thabet et al., 2018). Here, we identified a QTL and four candidate genes that were associated with seed germination capacity under HS through an integrated strategy combining SNP-trait association analysis, QTL mapping, and RNA-seq. These findings provide valuable gene resources for the genetic improvement of heat-tolerant seed germination capacity in watermelon.

Seed germination performance under abiotic stress is generally recognized as a polygenic quantitative trait (Thabet et al., 2018; Wang et al., 2011), and mainly influenced by the embryo genotype (Gu et al., 2014). Here, two watermelon varieties displayed markedly divergent germination phenotypes under HS. Under HS conditions, line 8R098 maintained a GP of 0.64 ± 0.11, GE of 0.43 ± 0.07, GI of 10.1 ± 1.34, RGP of 0.64 ± 0.11, and RGE of 0.43 ± 0.07, showcasing robust germination performance under HS (**Table 3**). In contrast, variety 8R122 and the F_1_ hybrid population derived from their cross failed to germinate normally under HS conditions (**Table 3**). Furthermore, the germination capacity under HS of seeds from individual F_2_ plants showed a bimodal distribution, with 54.2% of individuals having a 6-day germination rate below 0.1 and 22.14% having a rate of 1.0 (**Figure 1**). Furthermore, through defining high germination as RGP ≥ 0.6 and low germination as RGP < 0.6 under HS, we found that the segregation ratio of high to low individuals was roughly 1:3, suggesting control by one major recessive gene along with minor modifying genes. Besides, the contribution of maternal tissues to seed germination under HS should not be ignored, as they determine initial vigor and physical barriers. Future reciprocal crosses and near-isogenic lines could help separate maternal and embryonic contributions.

The identification of natural variation greatly improves our understanding of the genetic basis of agronomic traits (Liang et al., 2021). The QTLs associated with seed germination capacity under drought and salt stress have been identified in barley, rice, and lettuce, respectively (Thabet et al., 2018; Wang et al., 2011, Yoong et al., 2016). Here, we performed SNP-trait association analysis via whole-genome re-sequencing of 132 F_2_ individuals and two parental lines, and identified a QTL associated with seed germination capacity under HS on watermelon chromosome 10 (28.05~34.80 Mb) (**Figure 2**). In addition, this locus was validated by conventional QTL mapping, which narrowed the interval to 29.06~33.41 Mb (~4.35 Mb) (**Figure 3**). Unfortunately, given the limited size of the current F_2_ population, the mapping interval remains broad and contains dozens of protein-coding genes; therefore, we plan to enlarge the population for fine mapping in the future.

To prioritize causal candidates, we focused on HS-responsive genes with differential expression between high- and low-germination accessions under HS. RNA-seq analysis (**Supplementary Table S3**) identified 1,519 HS-responsive DEGs, enriched in plant hormone signal transduction and metabolic pathways (**Figure 3A** and **4**). We also detected 101 putative functional non-synonymous SNPs (nsSNPs) residing in 76 protein-coding genes within this interval. Intersection of these DEGs with the 76 nsSNP-harboring genes, followed by removal of genes with inconsistent expression patterns under HS, yielded four candidate genes, namely *Cla97C10G205480*, *Cla97C10G205850*, *Cla97C10G204760*, and *Cla97C10G203920*.

*Cla97C10G205480* (VARLMGL domain protein, methionine γ-lyase (MGL) homolog) carried a Gln232Glu (**Figure 4E**) substitution and may enhance HS tolerance through methionine catabolism to isoleucine, an osmoprotectant and energy source induced under abiotic stress during germination (Rébeillé et al., 2006; Hacham et al., 2023). *Cla97C10G205850* (putative pentatricopeptide repeat (PPR) protein) with an Arg445Trp change (**Figure 4E**) may be associated with organellar RNA metabolism and ABA signaling (Jiang et al., 2015; Li et al., 2023). *Cla97C10G204760* (chloroplast RETICULATA protein) has a Val129Ala substitution (**Figure 4E**) that may contribute to plastid membrane maintenance, critical for germination under HS (van Veen et al., 2025; Wang et al., 2023). *Cla97C10G203920* (clade A protein phosphatase 2C (PP2C)) harbored a Glu129Val alteration (**Figure 4E**) and was homologous to proteins previously reported to function as negative regulators of ABA signaling, affecting dormancy and germination (Nishimura et al., 2018; Ghanizadeh et al., 2025). Collectively, these four candidate genes may govern HS tolerance during seed germination via distinct pathways (**Supplementary Table S5**). Nonetheless, the present findings stem solely from omics-based inference. Further validation of target gene function should be carried out using overexpression and gene editing techniques.

In summary, we identified a QTL and four candidate genes associated with watermelon seed germination capacity under HS. These findings support breeding efforts for heat-tolerant germination and provide a foundation for elucidating the underlying molecular mechanisms.

## Data Availability

Sequencing data were submitted to CNSA (https://db.cngb.org/cnsa/, CNGBdb, accession CNP0007943). All relevant data are included in the article and **Supplementary Materials**, and further data can be requested from the corresponding author.
